# Limbic Encephalitis following Allogeneic Hematopoietic Stem Cell Transplantation

**DOI:** 10.1155/2022/4174755

**Published:** 2022-09-10

**Authors:** Silje Johansen, Jostein Kråkenes, C. A. Vedeler, Anette Margrethe Storstein, Håkon Reikvam

**Affiliations:** ^1^Department of Medicine, Haraldsplass Deaconess Hospital, N-5021 Bergen, Bergen, Norway; ^2^Department of Radiology, Haukeland University Hospital, N-5021 Bergen, Norway; ^3^Department of Clinical Medicine, University of Bergen, N-5021 Bergen, Norway; ^4^Department of Neurology, Haukeland University Hospital, N-5021 Bergen, Norway; ^5^Department of Medicine, Haukeland University Hospital, N-5021 Bergen, Bergen, Norway; ^6^Department of Clinical Science, University of Bergen, N-5021 Bergen, Norway

## Abstract

A woman with myelodysplastic syndrome (MDS) was treated with allogeneic hematopoietic stem cell transplantation (allo-HSCT). 65 days after the transplantation, she developed fatigue and central neurological symptoms. Clinical workup including magnetic resonance imaging (MRI) and cerebrospinal fluid (CSF) examination revealed findings suspicious for limbic encephalitis (LE), successfully treated with intravenous immunoglobulins and intravenous corticosteroids. Although a rare complication after allo-HSCT, physicians should be aware of neurological symptoms that develop throughout the transplantation course.

## 1. Introduction

The use of allogeneic hematopoietic stem cell transplantation (allo-HSCT) is increasing in most countries [1], since this treatment is becoming more reliable and more accessible. The treatment, on the other hand, is associated with a relatively high incidence of serious, and in worst case fatal, complications. Furthermore, neurological complications are rare, but a dreaded complication of allo-HSCT [2]. Reports support that a persistent neurological impairment occurs in many of those patients who suffer from neurological complications after allo-HSCT [3]. Herein, we present a patient who developed a serious neurological complication after allo-HSCT.

## 2. Case Presentation

**Figure 1:**
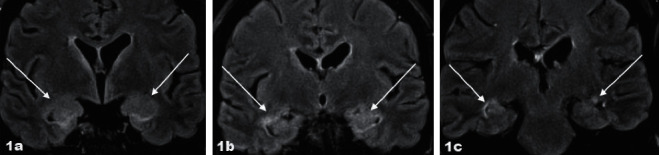
(a–c) Coronal FLAIR-MRI scan reveals slight enlargement and increased signal intensity of the amygdala and hippocampus bilaterally.

**Figure 2:**
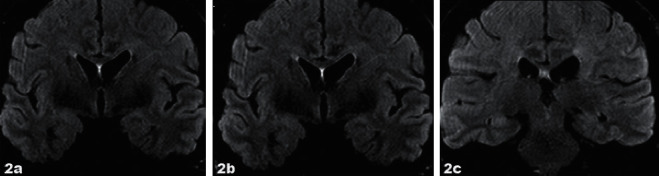
(a–c) Coronal FLAIR-MRI scan after treatment, four weeks later, was normal.

A 49-year-old previously healthy woman, with no history of mental illness or neurological abnormalities, was diagnosed with myelodysplastic syndrome (MDS) with fibrosis. She was initially treated with 5-azacitidine, while preparing for an allo-HSCT. Allo-HSCT was performed with reduced intensity conditioning (RIC) regimen with fludarabine, treosulfan, and anti-thymocyte globulin (ATG), before transplant with a matched unrelated donor (MUD), with both recipient and donor being positive for Epstein–Barr virus (EBV) and cytomegalovirus (CMV) IgG. She received cyclosporine A from the day before allo-HSCT and methotrexate the 1st, 3rd, and 6th day after allo-HSCT as prophylaxis towards graft versus host disease(GvHD). Already at day +21, she developed a generalized exanthema diagnosed as toxic epidermal necrolysis treated with intravenous immunoglobulin (IVIG) and methylprednisolone. The biopsy of the skin showed no signs of GvHD. She had a late engraftment, but at day +28, she had trilinear hematopoiesis and 99% donor chimerism and she showed no signs of acute GvHD (aGvHD). However, by day +65, she developed fatigue and altered mental level with confusion and seizures. She had low fever but normal C-reactive protein (CRP). The seizures were dominated by contractions lasting only seconds in face, neck, arm, and leg. However, an electroencephalogram (EEG) did not show seizure. Magnetic resonance imaging (MRI) demonstrated bilaterally increased signal intensity of the amygdala and hippocampus (Figures 1(a)–1(c)). Investigation of the cerebrospinal fluid (CSF) revealed increased number of leukocytes, 42 × 10^6^/L (reference <3), mononuclear pleocytosis, increased protein level 1.93 g/L (reference 0.15–0.50), and serum like IgG bands in isoelectric focusing of CSF. She was initially treated with broad-spectrum antibiotic and antiviral intravenous treatment, but these were discontinued as investigations for underlying viral or bacterial causes were negative, including human herpes virus-6 (HHV-6) in cerebrospinal fluid. Furthermore, as demonstrated in Table 1, 17 different standard onconeural and encephalitis autoantibodies were not detected in serum or CSF and antinuclear antibodies were not detected in serum. A computer tomography (CT) scan with intravenous contrast of the thoracic, abdominal, and pelvic regions had recently been performed with no signs of other malignancy. The clinical and radiological presentation were considered to be an autoimmune limbic encephalitis (LE) following allo-HSCT, due to cognitive impairment, seizures, MRI findings, and pleocytosis in the CSF. Accordingly, she was treated with intravenous corticosteroids, methylprednisolone 1000 mg for 5 days, and intravenous immunoglobulins for 5 days, total of 2 g/kg. She had an initial improvement, but the symptoms relapsed after 15 days, and the treatment was repeated and supplemented with tapering dose of per oral prednisolone for several weeks. The patient's symptoms went slowly in regression, and control MRI after four weeks was normal (Figures 2(a)–2(c)).

## 3. Discussion

The classical presentation and clinical findings of LE include rapidly progressive short-term memory loss, psychiatric symptoms, and seizures, combined with MRI findings of temporal lobe involvement, and CSF inflammatory abnormalities including detection of autoantibodies [4]. Our patient headlights the possibility of this rare complication post allo-HSCT and is a reminder that detection of autoantibodies in the CSF is not a prerequisite for the diagnosis of LE. Subacute development of short-term memory deficits is often typical for LE, although it was probably overlooked in the present case by other symptoms such as headache, irritability, sleep disturbance, delusions, hallucinations, and seizures [4]. Thermal sensations, piloerection, and paroxysmal dizziness might be symptoms resulting from focal seizure activity in LE [4]. Faciobrachial dystonic seizures are especially helpful diagnostically as they are nearly pathognomonic in a subtype of LE. A normal EEG during seizures should not eliminate the diagnosis, as coincident epileptiform discharges are seen only in the minority [4, 5]. Our patient did not have an EEG with typical findings that could correlate to the seizure, but an EEG with generalized low frequency activity supporting a diffuse central nerve system dysfunction.

LE is an inflammatory disease involving the mesial temporal lobes and the limbic structures, amygdala, and hippocampus. MRI often reveals abnormal high signal intensity on FLAIR and T2 weighted images in the mesial temporal lobes [6]. Typical MRI signal was found in our case. However, sometimes MRI may be normal, and in such cases, 18-fluorodeoxyglucose (18F-FDG) PET imaging has been reported to typically reveal mesial temporal lobe hypermetabolism [7].

Post-transplant acute/subacute LE has been described as a complication of allo-HSCT, occurring relatively early in the post-transplant period. Risk factors have been identified as heavily pretreating conditioning including ATG and corticosteroids treatment [8].

Neurological complications after allo-HSCT can be classified according to etiology or clinical presentation as listed in Table 2 [3]. The etiology of LE could be divided in infectious and non-infectious causes, where the former often has been related to HHV-6 reactivation [9]. However, without evidence of infectious origin, immunological mechanisms should be considered. When first-line therapy with steroids, immunoglobulins, and/or plasma exchange fails, one converts to second-line immunotherapy. Second-line immunotherapy includes usually monoclonal antibodies (mAbs) directed at B-cells such as the anti-CD20 antibody rituximab [10]. Our patient responded to corticosteroid and immunoglobulin therapy, although it had to repeated. We suggest that a rapid and circumstantiated diagnosis of autoimmune encephalitis, herein limbic encephalitis, could anticipate specific treatments and influence the rate or the extent of long-term disabilities [3].

## Figures and Tables

**Table 1:** All autoantibodies tested in serum and spinal fluid in our patient (all were below reference values).

Onconeuronal and encephalitis autoantibodies in spinal fluid	Anti LGI 1, anti CASPR2, anti gaba *R* b1/2, anti DPPX, anti-GluR type AMPA½, anti GluR type NMDA, anti Tr, anti Zic4, anti GAD 65, antiSOX1, anti Ma2, antiMa1, anti CRMP5, anti Amphiphy, anti Yo, anti Ri, anti Hu

Onconeuronal and encephalitis autoantibodies in serum	Anti LGI 1, anti CASPR 2, anti gaba *R* b1/2, anti DPPX, anti-GluR type AMPA ½, anti GluR type NMDA, anti MOG, aquaporin, anti Tr, anti Zic4, anti GAD 65, antiSOX1, anti Ma2, antiMa1, anti CRMP5, anti amphiphy, anti Yo, anti Ri, anti Hu

Antinuclear antibodies and antineutrophil cytoplasmic antibodies in serum	SM Ig *G*, RNP Ig *G*, SS-A Ig *G*, SS-B Ig *G*, Jo1IgG, Scl-70 Ig *G*, Centromer Ig *G*, dsDNA Ig *G*, anti -CRT, ribosomal P Ig *G*, SM RNP Ig *g*, anti Pr 3, Anti-MPO

**Table 2:** Neurological complication seen after allogeneic hematopoietic stem cell transplantation.

Classified neurological complications based on etiology	Drug-related
	Metabolic
	Infectious
	Cerebrovascular
	Immune/inflammatory
	CNS relapse of underlying disease
	Secondary CNS malignancy
	Undefined

Classified central nervous system neurological complications based on clinical disease	Focal epileptic seizure or general epileptic seizure
	Cranial neuropathy
	PRES^*∗*^
	Encephalopathy
	PML^*∗*^
	Meningoencephalitis or encephalitis
	Meningitis (leukemic, infectious), cerebritis, or ventriculitis
	Autoimmune encephalitis
	PALE^*∗*^
	ADEM^*∗*^
	LETM^*∗*^
	Optic neuritis
	Short myelitis
	Subarachnoid hemorrhage
	TA-TMA^*∗*^
	TIA^*∗*^
	NHL^*∗*^ relapse
	CNS-PTLD^*∗*^
	Cognitive impairment

^*∗*^PRES, posterior reversible encephalopathy syndrome, PALE, post-transplant acute limbic encephalitis, PML, progressive multifocal leukoencephalopathy, TA-TMA, transplant-associated thrombotic microangiopathy, TIA, transient ischemic attack, ADEM, acute disseminated encephalomyelitis, LETM, longitudinal extensive transverse myelitis, CNS-PTLD, central nervous system EBV-related post-transplant lymphoproliferative disorder, and NHL, non-Hodgkin lymphoma.

## Data Availability

No data were used to support this study.
